# The Dipeptidyl Peptidase-4 Inhibitor Linagliptin Directly Enhances the Contractile Recovery of Mouse Hearts at a Concentration Equivalent to that Achieved with Standard Dosing in Humans

**DOI:** 10.3390/ijms21165756

**Published:** 2020-08-11

**Authors:** Sri Nagarjun Batchu, Veera Ganesh Yerra, Youan Liu, Suzanne L. Advani, Thomas Klein, Andrew Advani

**Affiliations:** 1 Keenan Research Center for Biomedical Science and Li Ka Shing Knowledge Institute, St. Michael’s Hospital, Toronto, ON M5B 1T8, Canada; srinagarjun.batchu@unityhealth.to (S.N.B.); veeraganesh.yerra@unityhealth.to (V.G.Y.); youan.liu@unityhealth.to (Y.L.); suzanne.advani@unityhealth.to (S.L.A.); 2 Department of Cardiometabolic Diseases Research, Boehringer Ingelheim Pharma GmbH & Co. KG, 88397 Biberach, Germany; thomas_1.klein@boehringer-ingelheim.com

**Keywords:** DPP-4 inhibitor, linagliptin, Langendorff, nitric oxide, ischemia reperfusion injury

## Abstract

Despite a similar mechanism of action underlying their glucose-lowering effects in type 2 diabetes, dipeptidyl peptidase-4 (DPP-4) inhibitors have diverse molecular structures, raising the prospect of agent-specific, glucose-independent actions. To explore the issue of possible DPP-4 inhibitor cardiac heterogeneity, we perfused different DPP-4 inhibitors to beating mouse hearts ex vivo, at concentrations equivalent to peak plasma levels achieved in humans with standard dosing. We studied male and female mice, young non-diabetic mice, and aged diabetic high fat diet-fed mice and observed that linagliptin enhanced recovery after ischemia-reperfusion, whereas sitagliptin, alogliptin, and saxagliptin did not. DPP-4 transcripts were not detected in adult mouse cardiomyocytes by RNA sequencing and the addition of linagliptin caused ≤0.2% of cardiomyocyte genes to be differentially expressed. In contrast, incubation of C166 endothelial cells with linagliptin induced cell signaling characterized by phosphorylation of Akt and endothelial nitric oxide synthase, whereas the nitric oxide (NO) donor, S-nitroso-N-acetylpenicillamine increased serine 16 phosphorylation of the calcium regulatory protein, phospholamban in cardiomyocytes. Furthermore, linagliptin increased cardiomyocyte cGMP when cells were co-cultured with C166 endothelial cells, but not when cardiomyocytes were cultured alone. Thus, at a concentration comparable to that achieved in patients, linagliptin has direct effects on mouse hearts. The effects of linagliptin on cardiomyocytes are likely to be either off-target or indirect, mediated through NO generation by the adjacent cardiac endothelium.

## 1. Introduction

Dipeptidyl peptidase-4 (DPP-4) inhibitors are commonly used to lower glucose in Type 2 diabetes, although within the class they are quite diverse in their chemical structures [1]. This has raised the prospect of possible agent-specific differences in their effects, a possibility that is supported by heterogeneous heart failure outcomes observed in the cardiovascular safety trials that have been reported to date [2]. Linagliptin [3] and sitagliptin [4], for instance, were each associated with neutral effects on heart failure, whereas saxagliptin unexpectedly increased hospitalization for heart failure [5] and alogliptin numerically, but non-significantly, increased heart failure hospitalization [6]. However, although clinical trials have raised the question of DPP-4 inhibitor cardiac heterogeneity, they are unlikely to resolve it given the size of the undertaking that a clinical trial head-to-head comparison would require.

In a recent study of the cardiac effects of DPP-4 inhibition, we observed that mice fed the DPP-4 inhibitor linagliptin in chow exhibited enhanced recovery of cardiac contractile function following ischemia reperfusion [7]. In the present study, we set out to determine whether DPP-4 inhibitors themselves can directly affect cardiac contractility and, if so, whether there are within class differences in this effect. To facilitate the comparison, we perfused different DPP-4 inhibitors to isolated mouse hearts at concentrations equivalent to their peak plasma concentrations (C_max_) achieved with standard dosing in humans. Cognizant that sex differences have historically been overlooked in pre-clinical experimentation [8], we studied both male and female mice and we performed the head-to-head comparison under non-diabetic conditions and in the setting of comorbidity (aging, diabetes and high fat diet).

## 2. Results

### 2.1. When Perfused at A Concentration Equivalent to Human C_max_, Linagliptin Enhances the Contractile Recovery of Mouse Hearts after Ischemia Reperfusion

**Figure 1:**
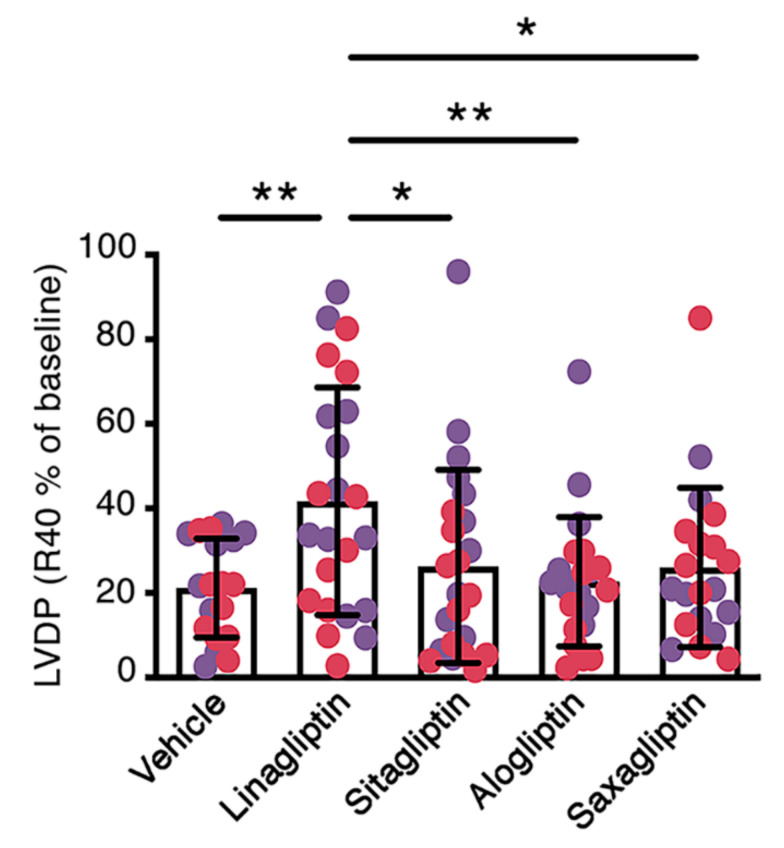
Perfusion of the hearts of young mice with linagliptin at human C_max_ equivalent enhances contractile recovery after ischemia reperfusion injury. Percentage recovery of left ventricular developed pressure 40 min after reperfusion (%LVDP R40) in the hearts of male (purple circles) and female (red circles) C57BL/6 mice aged 7–8 weeks perfused with vehicle (Krebs Henseleit buffer, KHB; *n* = 19) or KHB supplemented with 9 nmol/L linagliptin (*n* = 23), 817 nmol/L sitagliptin (*n* = 23), 110 ng/mL alogliptin (*n* = 22) or 24 ng/mL saxagliptin (*n* = 20). Values are mean ± S.D.. *p < 0.05, **p < 0.01 by one-way ANOVA followed by Fisher least significant difference post hoc test.

In our first experiments, we perfused in the Langendorff mode [7] the hearts of young adult, non-diabetic, male and female C57BL/6 mice with vehicle (Krebs Henseleit Buffer (KHB)) either alone or supplemented with linagliptin, sitagliptin, alogliptin, or saxagliptin at concentrations equivalent to peak plasma levels reported in humans with standard dosing (C_max_) (see Materials and Methods). Left ventricular developed pressure (LVDP), maximum and minimum dP/dt and heart rate at baseline and 40 min after reperfusion (following 20 min ischemia; R40) are shown in Table 1. Whereas baseline LVDP did not differ across the study groups, LVDP at R40 was significantly higher in linagliptin-perfused hearts than hearts perfused with KHB, sitagliptin, alogliptin, or saxagliptin (Table 1). Similarly, the percent change in LVDP from baseline (%LVDP R40) was higher in mouse hearts perfused with linagliptin than in the other study groups (Figure 1). dP/dt_max_ and dP/dt_min_ were numerically higher and lower, respectively, in linagliptin-perfused hearts than vehicle-perfused hearts. However, these differences did not achieve significance in multiple groups comparison (Table 1). Heart rate did not differ across the study groups (Table 1). Graphs separating these parameters into those for only male and female mice are shown in Supplementary Materials Figures S1 and S2.

### 2.2. Linagliptin Enhances the Contractile Recovery of The Hearts of Aged, Diabetic High Fat Diet-Fed Mice

Next, we asked whether the direct cardiac effects of DPP-4 inhibitors may be influenced by the metabolic milieu. Accordingly, we studied the hearts of aged, diabetic, high fat diet-fed (DM-HFD) mice. At the end of the study period, as expected, both body weight and blood glucose [9] were higher in male DM-HFD mice than female DM-HFD mice (mean body weight: male DM-HFD mice 38 ± 8 g, female DM-HFD mice 28 ± 4 g, *p* < 0.0001; mean blood glucose: male DM-HFD mice 18.1 ± 6.9 mmol/L, female DM-HFD mice 13.0 ± 4.3 mmol/L, *p* < 0.0001). Hearts were then extracted from DM-HFD mice and perfused in the Langendorff mode with sodium pyruvate-supplemented KHB (see Materials and Methods) in the presence or absence of the DPP-4 inhibitors linagliptin, sitagliptin, alogliptin, or saxagliptin at concentrations equivalent to human C_max_ as already described. In these experiments, we also decided to include the active metabolite of saxagliptin, 5-hydroxy saxagliptin, and GLP-1 itself.

**Figure 2:**
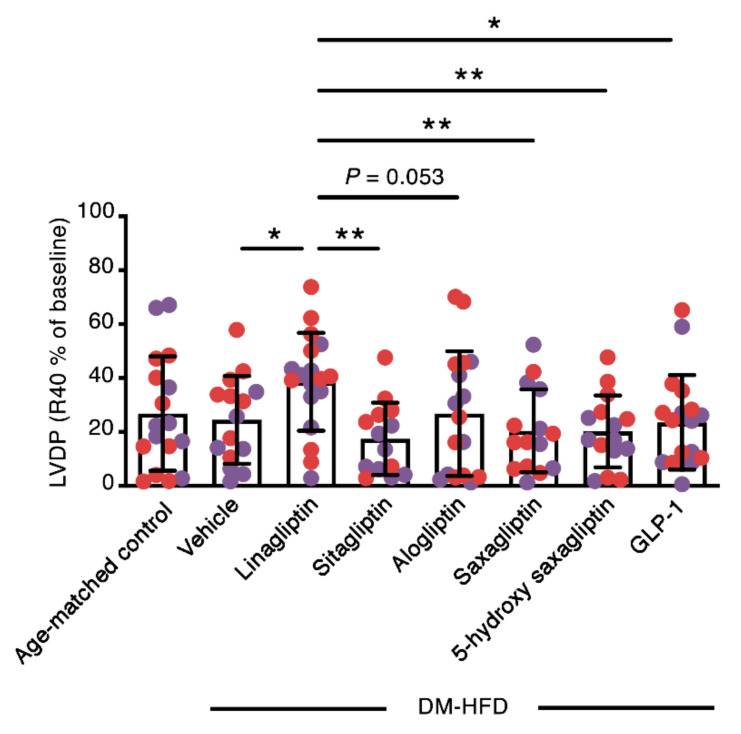
Perfusion with linagliptin at human C_max_ equivalent enhances the contractile recovery of the hearts of aged, diabetic high fat diet-fed (DM-HFD) mice after ischemia reperfusion injury. Percentage recovery of left ventricular developed pressure 40 min after reperfusion (%LVDP R40) in the hearts of male (purple circles) and female (red circles) C57BL/6 mice fed a high fat diet for 12 weeks, before receiving an intraperitoneal injection of streptozotocin (90 mg/kg) and fed a high fat diet for a further 14 weeks. Hearts were perfused with vehicle (sodium pyruvate (2 mmol/L) supplemented Krebs Henseleit buffer, KHB; *n* = 15) or sodium pyruvate supplemented KHB with 9 nmol/L linagliptin (*n* = 18), 817 nmol/L sitagliptin (*n* = 14), 110 ng/mL alogliptin (*n* = 17) or 24 ng/mL saxagliptin (*n* = 15), 47 ng/mL 5-hydroxy saxagliptin (*n* = 15) or 10 nmol/L GLP-1 (*n* = 18). Age-matched control (*n* = 17). Values are mean ± S.D.. *p < 0.05, **p < 0.01 by one-way ANOVA followed by Fisher least significant difference post hoc test.

Mean baseline LVDP varied between 63 cmH_2_O (for 5-hydroxy saxagliptin) and 103 cmH_2_O (for linagliptin) amongst the study groups, although these differences did not achieve statistical significance (Table 2). In contrast, like in the hearts of young, non-diabetic mice, both absolute LVDP 40 min after reperfusion (R40; Table 2) and the percentage change in LVDP (%LVDP R40; Figure 2) were higher in DM-HFD mouse hearts perfused with linagliptin than the hearts of DM-HFD mice perfused with vehicle, sitagliptin, alogliptin, or saxagliptin. Neither 5-hydroxy saxagliptin nor GLP-1 enhanced contractile recovery, whereas minimum dP/dt was higher in 5-hydroxy saxagliptin-perfused hearts than in DM-HFD hearts perfused with vehicle or linagliptin (Table 2). Supplementary Materials Figures S3 and S4 show graphs for the ex vivo hemodynamic parameters in DM-HFD mice separated for males and females, respectively.

### 2.3. Linagliptin Induces Phosphorylation of the Cardiomyocyte Ca^2+^ Handling Protein Phospholamban

**Figure 3:**
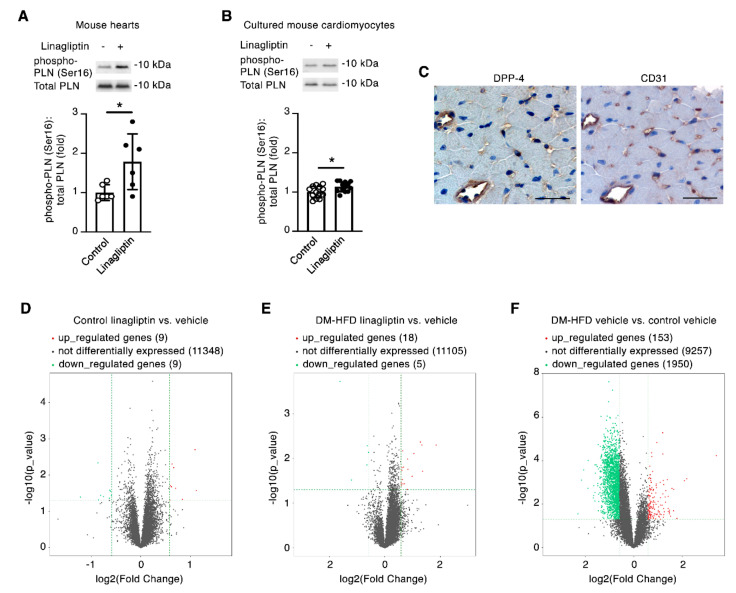
At human C_max_ equivalent, linagliptin indirectly increases Ser16 phosphorylation of the Ca^2+^ handling protein phospholamban in cardiomyocytes. (**A**) Immunoblotting for serine 16 phosphorylated phospholamban (PLN(Ser16)) in heart homogenates of young, non-diabetic adult mice perfused with Krebs Henseleit buffer (KHB) or KHB supplemented with 9 nmol/L linagliptin and subjected to 20 min no-flow ischemia followed by 40 min reperfusion (*n* = 6/group). (**B**) Immunoblotting primary cultured mouse cardiomyocytes for PLN(Ser16) under control conditions or after incubation with 9 nmol/L linagliptin for 5 min (*n* = 13/condition). (**C**) Serial cut sections from a C57BL/6 mouse heart immunostained for DPP-4 or CD31 showing predominant DPP-4 expression in CD31+ cells indicative of endothelial expression (*n* = 4). Scale bar = 50 µm. (**D**–**F**) Volcano plots of differentially expressed genes determined by RNA sequencing of adult mouse cardiomyocytes derived from young, non-diabetic, non-high fat diet-fed mice (D; *n* = 5/condition) or aged, diabetic, high fat diet-fed (DM-HFD) mice (E; *n* = 3/condition) exposed to linagliptin (9 nmol/L) or vehicle for 24 h. (**F**) Volcano plot of differentially expressed genes in vehicle-treated cardiomyocytes derived from DM-HFD mice (*n* = 3) and control mice (*n* = 5). Values are mean ± S.D.. *p < 0.05 by two-tailed Student’s *t*-test.

Having repeatedly observed that regardless of sex or comorbidity, linagliptin enhanced the recovery of contractile function of mouse hearts subjected to ischemia reperfusion injury, we next set out to explore how this effect may occur. The Ca^2+^ handling protein phospholamban (PLN) plays a pivotal role in regulating cardiomyocyte contractility. In its unphosphorylated form, PLN restricts cardiac contractility by limiting the affinity of SERCA2a for Ca^2+^ whereas, upon its Ser16 phosphorylation, PLN no longer restricts the SERCA2a pump, resulting in positive inotropic and lusitropic effects [10]. By immunoblotting, we observed phospho-PLN(Ser16) levels to be increased in linagliptin-perfused hearts in comparison to mouse hearts perfused with KHB (Figure 3A). To determine whether linagliptin (at human C_max_ equivalent) can directly induce PLN(Ser16) phosphorylation, we isolated primary cultured adult mouse cardiomyocytes and incubated the cells in the presence or absence of linagliptin. In this experiment, we observed a marginal, albeit statistically significant, increase in mouse cardiomyocyte phospho-PLN(Ser16) levels with linagliptin (Figure 3B). However, by immunostaining (and consistent with previous reports [11]), we found that DPP-4 protein expression was predominantly restricted to the endothelia of mouse hearts (Figure 3C). We isolated cardiomyocytes from adult C57BL/6 mice and DM-HFD mice and performed RNA sequencing after exposure of the cells to linagliptin or vehicle for 24 h. By performing differential gene expression analysis [12] (fold change cut off 1.5; *p* value ≤ 0.05; fragments per kilobase of transcript per million mapped reads (FPKM) ≥ 0.5 mean in one group), we observed that very few genes were differentially expressed in cardiomyocytes exposed to linagliptin. Indeed, in either cardiomyocytes from non-diabetic, non-high fat diet-fed (control) mice, or DM-HFD mice, the expression of ≤0.2% of genes was altered with linagliptin exposure (Figure 3D,E and Supplementary Materials Tables S1–S4). In contrast, ~18.5% of genes were differentially expressed in a comparison of the cardiomyocyte transcriptomes between control mice and DM-HFD mice (Figure 3F). Furthermore, at the depth of our RNA sequencing (40 million paired-end reads), we did not detect DPP-4 transcripts in primary cultured adult mouse cardiomyocytes.

### 2.4. Linagliptin Directly Induces Intracellular Signaling in Endothelial Cells and Indirectly Induces Intracellular Signaling in Cardiomyocytes

**Figure 4:**
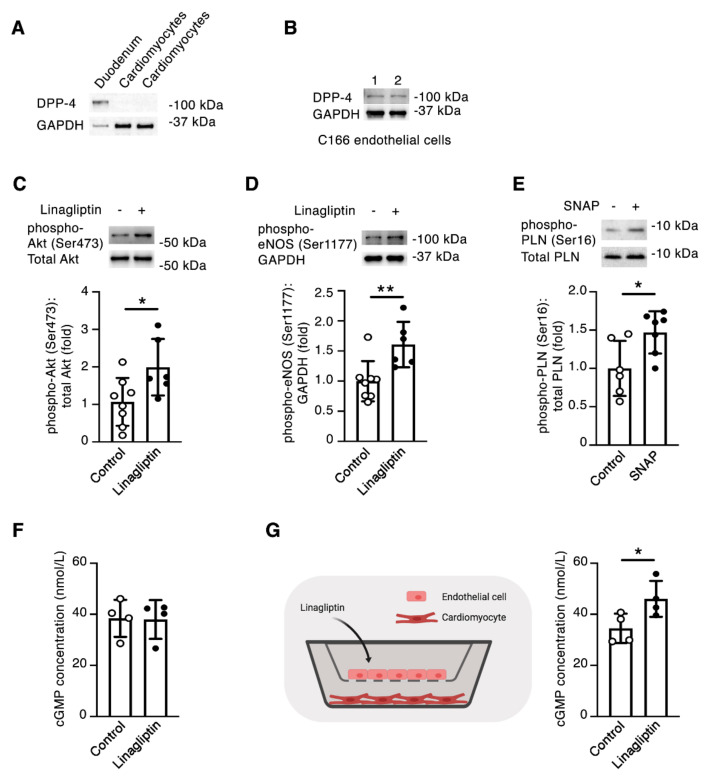
Linagliptin promotes NO-mediated endothelial-cardiomyocyte communication. (**A**) Immunoblotting showing absence of DPP-4 in primary cultured mouse cardiomyocytes (mouse duodenum = positive control). (**B**) Immunoblotting C166 endothelial cell lysates for DPP-4 (1 and 2 are replicates). (**C** and **D**) Immunoblotting C166 mouse endothelial cells for phospho-Akt (Ser473) (C) or phospho-eNOS (Ser1177) (D) after incubation with 9 nmol/L linagliptin for 5 min (control, *n* = 8, linagliptin, *n* = 6). (**E**) Immunoblotting adult mouse cardiomyocytes for PLN(Ser16) after exposure to 100 µmol/L S-nitroso-N-acetylpenicillamine (SNAP) for 5 min (vehicle, *n* = 6; SNAP, *n* = 7). (**F**) cGMP concentration in adult mouse cardiomyocytes under control conditions or after incubation with 9 nmol/L linagliptin for 5 min (*n* = 4/condition). (**G**) (Left) Co-culture experiment in which adult mouse cardiomyocytes were cultured in the presence of C166 endothelial cells in cell culture inserts before the addition of linagliptin. (Right) cGMP concentration in adult mouse cardiomyocytes co-cultured with C166 endothelial cells under control conditions or after incubation with 9 nmol/L linagliptin for 5 min. Values are mean ± S.D.. * *p* < 0.05, ** *p* < 0.01 by the two-tailed Student’s *t*-test.

Given the apparently robust effect of linagliptin in improving the ex vivo recovery of intact hearts, but the comparably minimal effect of linagliptin on cardiomyocytes themselves, we hypothesized that the DPP-4 inhibitor may thus mediate its effects on cardiomyocytes indirectly through actions in another cell-type, most likely endothelial cells. Through immunoblotting, we confirmed the absence of DPP-4 protein in isolated mouse cardiomyocytes (Figure 4A), but the presence of DPP-4 protein in the mouse C166 endothelial cell line (Figure 4B). We exposed C166 endothelial cells to linagliptin and observed increases in both the Ser473 phosphorylation of Akt (Figure 4C) and Ser1177 phosphorylation of eNOS (Figure 4D). Furthermore, treatment of primary cultured mouse cardiomyocytes with the nitric oxide (NO) donor, SNAP resulted in an ~50% increase in PLN(Ser16) phosphorylation (Figure 4E), comparable to the levels we had earlier observed in mouse hearts perfused with linagliptin (Figure 3A). The major target of NO signaling in the cardiovascular system is soluble guanylyl cyclase, which generates the second messenger cyclic guanosine monophosphate (cGMP) [13], that in turn facilitates cardiomyocyte PLN(Ser16) phosphorylation [14]. Accordingly, in our final experiments, we set out to determine whether linagliptin affects cardiomyocyte cGMP generation. When isolated cardiomyocytes were exposed to linagliptin at human C_max_ equivalent (9 nmol/L), we observed no change in cGMP concentrations (Figure 4F). In contrast, when we repeated the experiment with cardiomyocytes co-cultured in the presence of C166 endothelial cells in cell culture inserts, we observed an approximate 33% increase in cardiomyocyte cGMP with linagliptin (Figure 4G).

## 3. Discussion

Although there is structure-based rationale and indirect clinical trial evidence supporting the possibility of biological differences in the cardiac effects of different DPP-4 inhibitors, such a distinction has until now largely been conjectural in the absence of head-to-head comparison. In the present study, we took advantage of an ex vivo system to compare the cardiac effects of different DPP-4 inhibitors, observing that linagliptin consistently improved the recovery of contractile function after ischemia reperfusion injury. In exploring the mechanisms underlying this effect, we concluded that linagliptin has direct effects on mouse hearts at a concentration equivalent to that achieved in humans with standard dosing. However, whereas the cardiac actions of linagliptin result in phosphorylation of the Ca^2+^ handling protein PLN, they are most likely mediated in an indirect manner, plausibly through the actions of linagliptin on the cardiac endothelium.

The first two sub-studies herein described reported the findings of fairly large comparisons of different DPP-4 inhibitors when directly perfused to beating mouse hearts subjected to ischemia reperfusion. The Langendorff system that we utilized has two specific advantages for this comparison. First, it enabled us to examine the direct cardiac effects of the DPP-4 inhibitors isolated from broader neurohumoral effects that could occur through in vivo administration. Second, it allowed us to perform a head-to-head comparison by matching the different DPP-4 inhibitors using concentrations equivalent to the peak plasma levels achieved in patients with doses of the agents routinely employed in the clinic.

We began by comparing the DPP-4 inhibitors linagliptin, sitagliptin, alogliptin, and saxaglipin in the hearts of male and female mice and expanded our second sub-study to also include the active metabolite 5-hydroxy saxagliptin and the incretin hormone GLP-1 in the hearts of aged, diabetic high fat diet-fed mice. Regardless of mouse sex or comorbidity, we repeatedly observed improved recovery of LVDP after ischemia reperfusion in hearts perfused with linagliptin, an observation that was not recapitulated in the other experimental conditions. Although we observed a marginal increase in PLN(Ser16) phosphorylation by directly applying linagliptin to cardiomyocytes, we reasoned that this was most likely to be an off-target effect, noting that: (i) DPP-4 is primarily endothelial in its expression patterns in mouse hearts; (ii) DPP-4 protein was not detected in isolated cardiomyocytes by immunoblotting; (iii) DPP-4 transcripts in cardiomyocytes were not detected at the depth of our RNA sequencing; and (iv) linagliptin induced few gene expression changes in cardiomyocytes.

We speculated that an alternative means by which linagliptin affects cardiac contractility could be through its actions on the cardiac endothelium, which could in turn regulate cardiomyocyte function. Consistent with such a possibility, incubation of C166 endothelial cells with linagliptin resulted in Akt and eNOS phosphorylation whereas the NO donor, SNAP enhanced PLN(Ser16) phosphorylation in cardiomyocytes to levels similar to those seen in intact hearts perfused with linagliptin. Several previous studies have reported the activation of endothelial Akt/eNOS signaling by DPP-4 inhibitors including linagliptin [15,16,17]. In turn, NO produced by the endothelium can enhance cardiac contractility by promoting PLN(Ser16) phosphorylation in cardiomyocytes [13]. More specifically, NO increases cardiomyocyte cGMP levels that inhibit phosphodiesterase 3 (PDE3), preventing the inactivation of cyclic adenosine monophosphate (cAMP), and thus activating protein kinase A (PKA) [18], which phosphorylates PLN on serine residue 16 [19]. In our experiments, we observed an increase in cardiomyocyte cGMP with linagliptin when cells were co-cultured with C166 endothelial cells, but not when cardiomyocytes were exposed to linagliptin in isolation, supporting the assertion that linagliptin exerts its effects on cardiomyocytes through a signaling cascade initiated in the endothelium.

The role that NO plays in the heart can be both complex and conflicting [18,20]. Whereas low concentrations of NO are positively inotropic, at higher concentrations, NO induces higher levels of cGMP, which activate cGMP-dependent protein kinase (PKG). Among its actions, PKG inhibits voltage-dependent Ca^2+^ channels [20,21] and decreases myofilament Ca^2+^ responsiveness [22], actually impairing cardiac contractility. In addition, NO can interfere with mitochondrial respiration and at high concentrations, it can induce necrosis or apoptosis and affect platelet aggregation and the interaction of neutrophils with the endothelium [18]. Furthermore, the actions of NO in the heart are also influenced by the setting in which they occur (experimental or (patho)physiological), species, the presence of comorbidities, and drugs that affect NO release [18,23]. Studies conducted approximately 20 years ago reported that mice genetically deficient in eNOS paradoxically exhibit enhanced recovery following ischemia reperfusion, which has been attributed to inducible nitric oxide synthase (iNOS) superinduction or negation of peroxynitrite formation [24,25]. Accordingly, there may be circumstances in which DPP-4 inhibitor-induced NO production in the cardiac endothelium is not advantageous and could even potentially be deleterious.

A number of limitations in the present study warrant emphasis. For instance, although we observed differences in the cardiac effects of linagliptin in isolated perfused mouse hearts compared to the other DPP-4 inhibitors tested, the cause of this difference has not been defined. Comparative binding property studies indicate that each of the commonly available DPP-4 inhibitors bind in the same catalytic site of the enzyme [26,27]. However, there are differences in the subsite binding interactions within the drug class [26,27]. DPP-4 inhibitors can also differ substantially in their first order rate constant for dissociation of the protein-ligand complex (*k*_off_), target-binding being >1000 times longer for linagliptin than sitagliptin, for instance [28]. As a cell surface glycoprotein, DPP-4 interacts with a number of other proteins (including adenosine deaminase, fibronectin, collagen, and C-X-C chemokine receptor type 4 (CXCR4)) and it is thus conceivable that individual DPP-4 inhibitors could have unique effects based on the manner in which they sterically hinder these interactions. However, this possibility remains speculation and has not been examined in the present study. In addition, although we observed that linagliptin induces Akt/eNOS signaling in C166 endothelial cells at human C_max_ equivalent, the proximal events by which linagliptin induces this signaling cascade have not been resolved. It has been suggested that these events could be caused by linagliptin’s direct interference with the interaction between eNOS and caveolin-1, blocking the negative allosteric regulation of eNOS by caveolin-1 [17,29]. Downstream, whereas we focused on the effects of NO on cardiomyocyte PLN (Ser16) phosphorylation in mouse hearts and in primary cells, it should be recognized that improved recovery after ischemia reperfusion may also occur through NO-mediated improvements in coronary flow. Despite its limitations though, the present study does provide important new insights. In particular, use of the isolated heart perfusion system has enabled the demonstration that linagliptin, at a clinically relevant concentration, does have direct effects on mouse hearts irrespective of sex or comorbidity. This effect occurs without meaningful change in the cardiomyocyte transcriptome and may relate to the induction of endothelial signaling, which ultimately leads to the Ser16 phosphorylation of PLN in cardiomyocytes, enhancing cardiac contractility.

In summary, at least under the experimental conditions herein reported, DPP-4 inhibitors do have agent-specific direct cardiac effects. Linagliptin directly enhances the recovery of cardiac contractility after ischemia reperfusion injury at concentrations that are achieved in humans with standard dosing. These actions are likely to occur through the modulation of NO-regulated pathways.

## 4. Materials and Methods

### 4.1. Isolated Heart Perfusion Study 1

Hearts from male and female C57BL/6 mice (C57BL/6NCrl) aged seven to eight weeks (Charles River Laboratories, Senneville, Quebec, Canada) were perfused using a retrograde isolated perfusion technique [30]. Briefly, mice were anesthetized with an intraperitoneal injection of tribomoethanol (250 mg/kg) and hearts were isolated and mounted on a Radnoti Langendorff Constant Pressure Apparatus (AD Instruments, Colorado Springs, CO, USA). Hearts were perfused at constant pressure and with continuously oxygenated KHB maintained at 37 °C. KHB was supplemented with different DPP-4 inhibitors at the following concentrations for the duration of the perfusion, according to their reported dosage equivalent C_max_ in humans [31]: linagliptin 9 nmol/L (achieved with the 5 mg oral dosage in humans) [32], sitagliptin 817 nmol/L (achieved with the 100 mg oral dosage in humans) [33], alogliptin 110 ng/mL (achieved with the 25 mg oral dosage in humans without significant organ dysfunction) [34,35] and saxagliptin 24 ng/mL (achieved with the 5 mg oral dosage in humans) [31] (each from Boehringer Ingelheim Pharma GmbH & Co. KG, Biberach, Germany). Perfused hearts were stabilized for 40 min and then subjected to 20 min of global no-flow ischemia, followed by 40 min of reperfusion. Left ventricular developed pressure (LVDP), maximum and minimum dP/dt and heart rate were collected and analyzed using PowerLab 8/35 and LabChart Pro (AD Instruments).

### 4.2. Isolated Heart Perfusion Study 2

Male and female C57BL/6 mice (Charles River Laboratories) were placed on a high fat diet (45% kcal fat, 35% kcal carbohydrate, and 0.05% weight for weight cholesterol; Research Diets Inc., New Brunswick, NJ, USA). After 12 weeks, mice received a single intraperitoneal injection of streptozotocin (STZ; 90 mg/kg in 0.1 mol/L sodium citrate, pH 4.5). Diabetes was confirmed two weeks after STZ injection by blood glucose testing (One Touch UltraMini; LifeScan Canada Ltd., Burnaby, British Columbia, Canada) and mice that were not diabetic (blood glucose <12 mmol/L) received a second dose of STZ. Animals were continued on a high fat diet and were followed for a total of 26 weeks. Diabetic high fat diet-fed (DM-HFD) mice were then anesthetized and hearts perfused as already described. As we had observed in our previous work that the hearts of DM-HFD mice could exhibit poor recovery after 20 min no-flow ischemia [7], we supplemented the KHB with 2 mmol/L sodium pyruvate throughout the duration of the procedure [36]. Mouse hearts were perfused with buffer alone or buffer supplemented with 9 nmol/L linagliptin, 817 nmol/L sitagliptin, 11 ng/mL alogliptin, 24 ng/mL saxagliptin, 47 ng/mL 5-hydroxy saxagliptin (achieved with the 5 mg oral dose of saxagliptin in humans [37]) (Biomol GmbH, Hamburg, Germany) or 10 nmol/L glucagon-like peptide 1 (GLP-1) [38,39] (Biotrend Chemikalien GmbH, Köln, Germany).

All experimental procedures adhered to the guidelines of the Canadian Council on Animal Care and were approved by the St. Michael’s Hospital Animal Care Committee (ACC736, 7 March 2017).

### 4.3. Cell Culture

Adult primary cultured cardiomyocytes were isolated from mouse hearts following the protocol described by Ackers-Johnson et al [40] and C166 mouse yolk sac endothelial cells were from American Type Culture Collection (ATCC) (Manassas, VA, USA). Cells were incubated in the presence or absence of linagliptin (9 nmol/L) for 5 min for immunoblotting experiments and for 24 h for RNA sequencing. In addition, cardiomyocytes were incubated in the presence or absence of 100 µmol/L S-nitroso-*N*-acetylpenicillamine (SNAP) (Sigma-Aldrich, Oakville, Ontario, Canada) [41] for 5 min prior to immunoblotting. RNA isolation from cardiomyocytes was performed using TRIzol Reagent (Life Technologies, Carlsbad, CA, USA). For co-culture experiments, adult primary cultured cardiomyocytes and C166 cells were cultured separately on opposite sides of Millicell 6-well plate inserts with a 10 µm pore size PET membrane (MCRP06H48, EMD Millipore, Billerica, MA, USA) at a 1:2 density under control conditions or with culture media supplemented with 9 nmol/L linagliptin for 5 min. Cardiomyocyte cGMP concentrations were determined by direct immunoassay (ab65356, Abcam, Cambridge, MA, USA) according to the manufacturer’s instructions.

### 4.4. Immunoblotting

Immunoblotting was performed on mouse heart homogenates, isolated adult mouse cardiomyocytes or C166 endothelial cell lysates with the following antibodies: phosphorylated phospholamban (phospho-PLN) Ser16 1:1000 (A285) [42], total PLN 1:1000 (1D11) [43], DPP-4 1:1000 (AF954, R & D Systems, Minneapolis, MN, USA); phospho-Akt (Ser473) 1:1000 (#9271, Cell Signaling Technology, Danvers, MA, USA), total Akt 1:1000 (#9272, Cell Signaling Technology), phospho-endothelial nitric oxide synthase (phospho-eNOS) (Ser1177) 1:1000 (#07-428-I, Sigma-Aldrich), or GAPDH 1:1000 (#2118, Cell Signaling Technology). Densitometry was performed using ImageJ version 1.39 (National Institutes of Health, Bethesda, MD, USA).

### 4.5. Immunohistochemistry

Immunohistochemistry on serially cut mouse heart sections was performed with the following antibodies: DPP-4 1:50 dilution (AF954, R & D Systems), secondary antibody rabbit anti-goat 1:100 dilution (CLDB200, Cedarlane, Burlington, Ontario, Canada), CD31 1:100 (sc-1506-R, Santa Cruz Biotechnology, Dallas, TX, USA), secondary antibody Dako Envision+ System-HRP labeled polymer anti-rabbit (K4003, Agilent Technologies Canada Inc., Mississauga, Ontario, Canada), as previously described [44].

### 4.6. RNA Sequencing

RNA sequencing was performed using the 6G RNA Sequencing Service (150bp paired-end, 40 million reads) (Arraystar Inc., Rockville, MD, USA). In brief, mRNA was enriched using NEBNext Poly(A) mRNA Magnetic Isolation Module (New England Biolabs Inc., Ipswich, MA, USA) and RNA sequencing library preparation was performed using a KAPA Stranded RNA-Seq Library Prep Kit (Illumina, San Diego, CA, USA). Sequencing (150 cycles for both ends) was performed on an Illumina Novaseq 6000. Image analysis and base calling was performed using Solexa pipeline v1.8 (Off-Line Base Caller software, v1.8) and sequence quality was examined using FastQC software. Trimmed reads (trimmed 5’, 3’-adaptor bases using cutadaptor) were aligned to the reference genome (GRCm38) using Hisat2 software. The transcript abundances for each sample were estimated using StringTie [45] and the FPKM, differential gene expression and volcano plots were generated using R package Ballgown [12]. RNA sequencing data are available at Gene Expression Omnibus (accession no. GSE144796).

### 4.7. Statistics

Data are expressed as mean ± S.D. Statistical significance was determined by one-way ANOVA with a Fisher least significant difference test for comparison of multiple groups or a two-tailed Student’s *t*-test for comparison between two groups. Statistical analyses were performed using GraphPad Prism 8 for macOS (GraphPad Software Inc., San Diego, CA, USA). A *p* value < 0.05 was considered statistically significant.

## Figures and Tables

**Table 1:** Effect of different DPP-4 inhibitors, administered at human C_max_ equivalent, on the ex vivo function of hearts of non-diabetic young mice at baseline and 40 min after ischemia perfusion (R40).

		LVDP (cmH_2_O)		dP/dt_max_ (cmH_2_O × ms^−1^)		dP/dt_min_ (cmH_2_O × ms^−1^)		Heart Rate (bpm)	
	*n*	Baseline	R40	Baseline	R40	Baseline	R40	Baseline	R40
Vehicle	19	94 ± 32	21 ± 16	3493 ± 1527	1268 ± 904	−2821 ± 979	−1127 ± 746	278 ± 86	348 ± 89
Linagliptin	23	95 ± 35	42 ± 32 ^a^	3957 ± 1957	2426 ± 2025	−2762 ± 1080	−1782 ± 1207	306 ± 77	377 ± 90
Sitagliptin	23	84 ± 29	21 ± 16 ^b^	4058 ± 2317	1667 ± 1188	−2706 ± 1063	−1335 ± 840	277 ± 95	340 ± 89
Alogliptin	22	88 ± 43	20 ± 18 ^b^	4134 ± 2722	1492 ± 1077	−2930 ± 1486	−1289 ± 908	294 ± 97	331 ± 87
Saxagliptin	20	90 ± 36	23 ± 18 ^c^	3898 ± 2522	1765 ± 1632	−2765 ± 1158	−1297 ± 894	300 ± 109	332 ± 121

Values are mean ± S.D.. ^a^ *p* < 0.01 vs. vehicle, ^b^ *p* < 0.001 vs. linagliptin, ^c^ *p* < 0.01 vs. linagliptin by one-way ANOVA followed by Fisher least significant difference post hoc test. bpm = beats per minute.

**Table 2:** Effect of different DPP-4 inhibitors, 5-hydroxy saxagliptin, or GLP-1 on the ex vivo function of hearts of aged diabetic high fat diet-fed mice (DM-HFD) at baseline and 40 min after ischemia perfusion (R40).

		LVDP (cmH_2_O)		dP/dt_max_ (cmH_2_O × ms^−1^)		dP/dt_min_ (cmH_2_O × ms^−1^)		Heart Rate (bpm)	
	*n*	Baseline	R40	Baseline	R40	Baseline	R40	Baseline	R40
Age-matched control + Vehicle	17	101 ± 30	25 ± 19	3777 ± 1913	1274 ± 1011	−2600 ± 960	−1295 ± 1240	275 ± 101	337 ± 98
DM-HFD + Vehicle	15	90 ± 27	21 ± 14	3433 ± 1021	1266 ± 796	−2541 ± 604	−1152 ± 692	295 ± 83	293 ± 139
DM-HFD + Linagliptin	18	103 ± 22	38 ± 16 ^ab^	3951 ± 1856	1920 ± 1295	−2763 ± 946	−1639 ± 1521	288 ± 87	327 ± 86
DM-HFD + Sitagliptin	14	81 ± 39	14 ± 11 ^c^	3139 ± 1894	1065 ± 870	−2156 ± 1143	−913 ± 760	311 ± 91	320 ± 108
DM-HFD + Alogliptin	17	83 ± 28	20 ± 19 ^d^	3422 ± 2024	1212 ± 949	−2483 ± 1361	−1313 ± 1475	297 ± 114	362 ± 148
DM-HFD + Saxagliptin	15	81 ± 15	15 ± 11 ^c^	3219 ± 1620	1156 ± 1099	−2251 ± 993	−957 ± 842	278 ± 94	321 ± 135
DM-HFD + 5-hydroxy saxagliptin	15	69 ± 54	14 ± 11 ^ac^	2578 ± 2275	805 ± 871	−1744 ± 1393 ^aef^	−672 ± 732	237 ± 73	317 ± 106
DM-HFD + GLP-1	18	96 ± 39	21 ± 17 ^e^	3929 ± 1486	1400 ± 937	−2981 ± 839 ^gh^	−1287 ± 758	311 ± 93	346 ± 91

Values are mean ± S.D. ^a^ *p* < 0.05 vs. control + vehicle, ^b^ *p* < 0.01 vs. DM-HFD + vehicle, ^c^ *p* < 0.0001 vs. DM-HFD + linagliptin, ^d^ *p* < 0.001 vs. DM-HFD + linagliptin, ^e^ *p* < 0.01 vs. DM-HFD + linagliptin, ^f^ *p* < 0.05 vs. DM-HFD + vehicle, ^g^ *p* < 0.05 vs. DM-HFD + sitagliptin, ^h^ *p* < 0.01 vs. DM-HFD + 5-hydroxy saxagliptin by 1-way ANOVA followed by Fisher least significant difference post hoc test. bpm = beats per minute.

## Supplementary Materials

The following are available online at https://www.mdpi.com/1422-0067/21/16/5756/s1.
