# Supplementary material for: The Dipeptidyl Peptidase-4 Inhibitor Linagliptin Directly Enhances the Contractile Recovery of Mouse Hearts at a Concentration Equivalent to that Achieved with Standard Dosing in Humans
Source: Int J Mol Sci. 2020 Aug 11;21(16):5756. doi: 10.3390/ijms21165756 (PMC7460951; doi:10.3390/ijms21165756)
Supplement: Supplementary file 1 [file ijms-21-05756-s001.pdf]

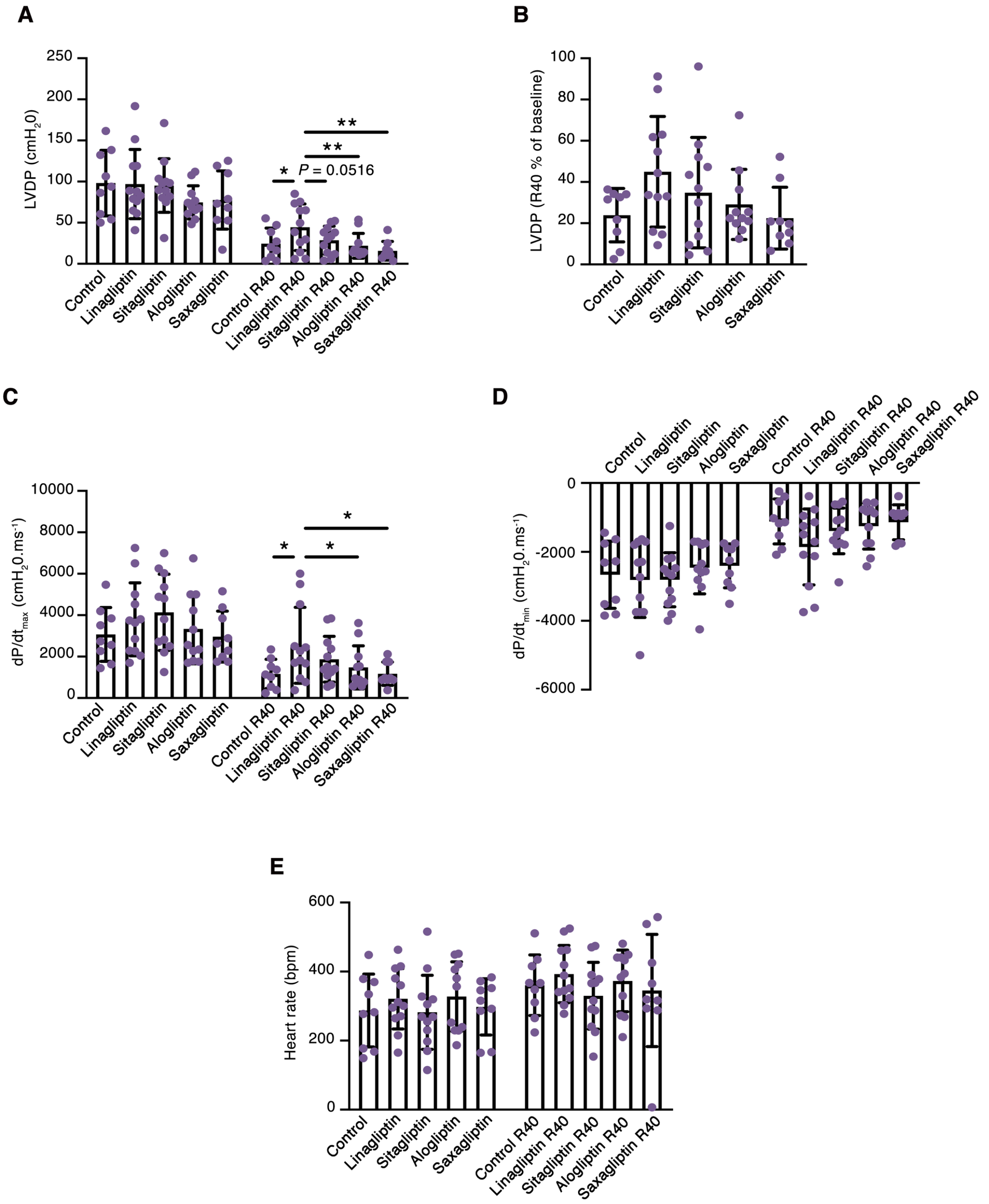

Supporting Information Figure 1. Effect of different DPP-4 inhibitors, administered at human C_{max} equivalent, on the ex vivo function of hearts of non-diabetic young male mice at baseline and 40 minutes after ischemia perfusion (R40). (A) Left ventricular developed pressure (LVDP). (B) Percentage recovery of LVDP 40 minutes after reperfusion (%LVDP R40). (C) $dPdt_{max}$. (D) $dPdt_{min}$. (E) Heart rate. bpm = beats per minute. Values are mean ± S.D.. Control (n=9), linagliptin (n=12), sitagliptin (n=12), alogliptin (n=11), saxagliptin (n=9). *P < 0.05, **P < 0.01 by 1-way ANOVA followed by Fisher least significant difference post hoc test.

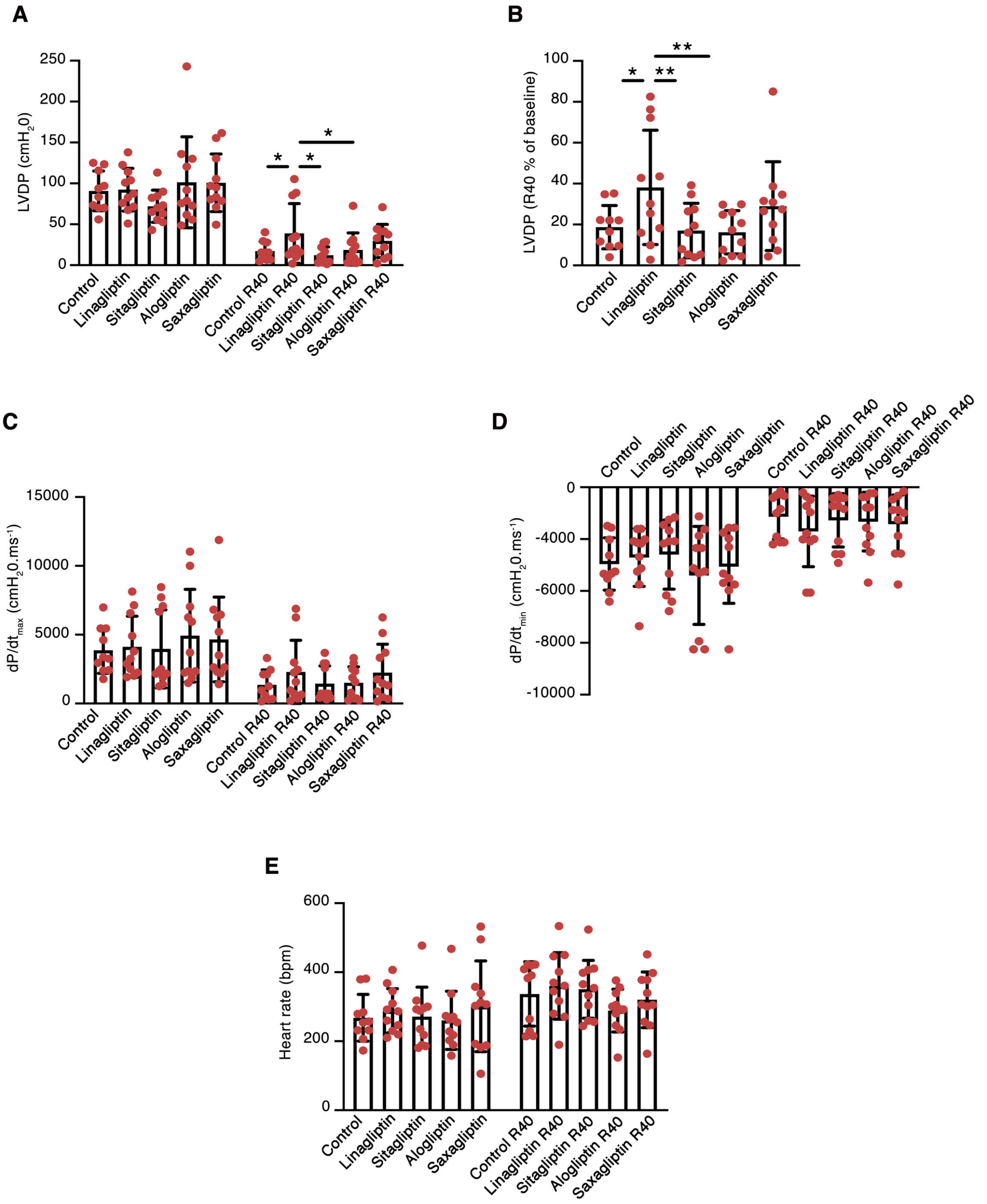

Supporting Information Figure 2. Effect of different DPP-4 inhibitors, administered at human C_{max} equivalent, on the ex vivo function of hearts of non-diabetic young female mice at baseline and 40 minutes after ischemia perfusion (R40). (A) Left ventricular developed pressure (LVDP). (B) Percentage recovery of LVDP 40 minutes after reperfusion (%LVDP R40). (C) $dPdt_{max}$. (D) $dPdt_{min}$. (E) Heart rate. bpm = beats per minute. Values are mean ± S.D.. Control (n=10), linagliptin (n=11), sitagliptin (n=11), alogliptin (n=11), saxagliptin (n=11). *$P < 0.05$, **$P < 0.01$ by 1-way ANOVA followed by Fisher least significant difference post hoc test.

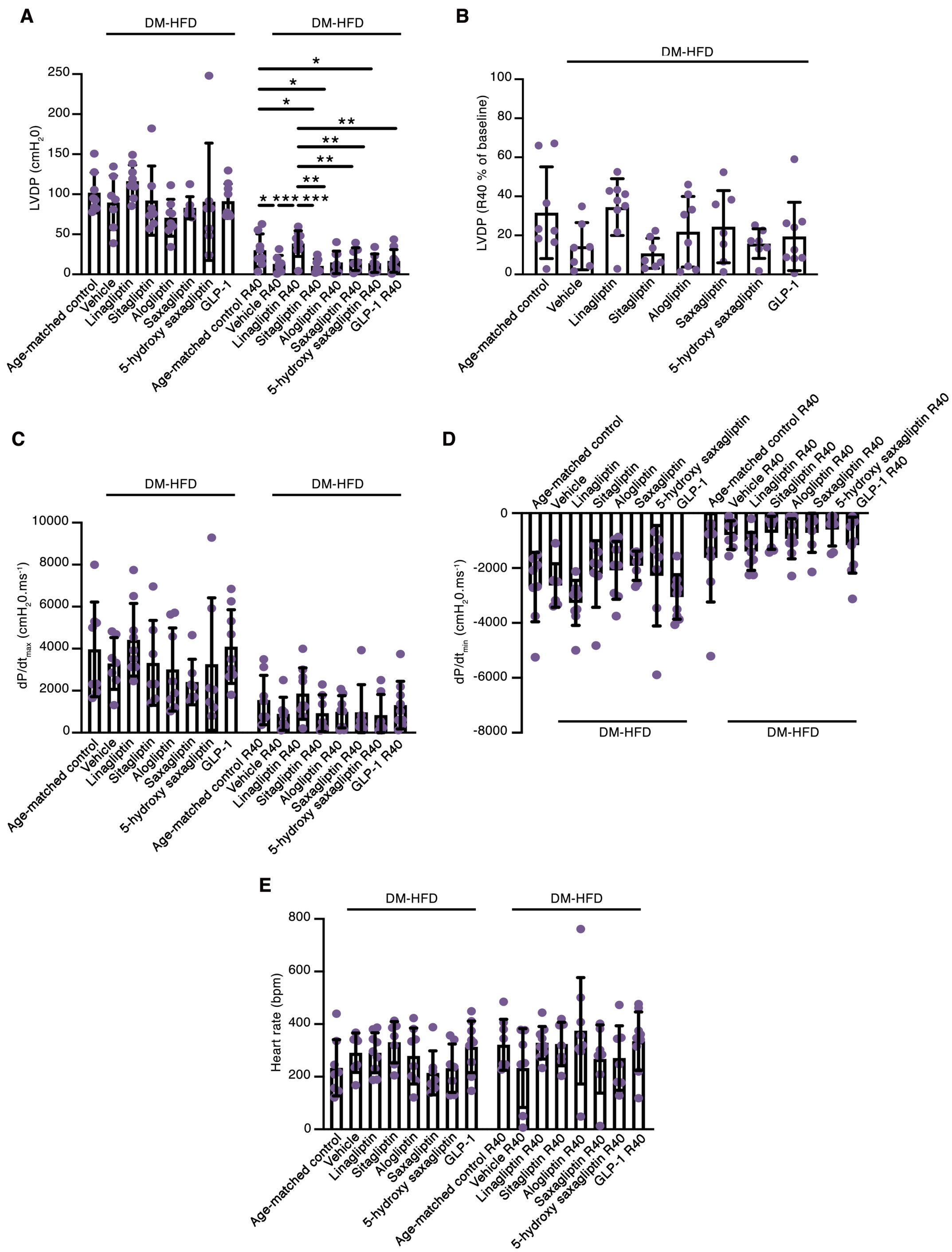

Supporting Information Figure 3. Effect of different DPP-4 inhibitors, administered at human C_{max} equivalent, on the ex vivo function of aged, diabetic high fat diet-fed (DM-HFD) male mice at baseline and 40 minutes after ischemia perfusion (R40). (A) Left ventricular developed pressure (LVDP). (B) Percentage recovery of LVDP 40 minutes after reperfusion (%LVDP R40). (C) $dPdt_{max}$. (D) $dPdt_{min}$. (E) Heart rate. bpm = beats per minute. Values are mean ± S.D.. Age-matched control (n=9), vehicle (n=8), linagliptin (n=9), sitagliptin (n=7), alogliptin (n=9), saxagliptin (n=8), 5-hydroxy saxagliptin (n=8), GLP-1 (n=9). *P < 0.05, **P < 0.01, ***P < 0.001 by 1-way ANOVA followed by Fisher least significant difference post hoc test.

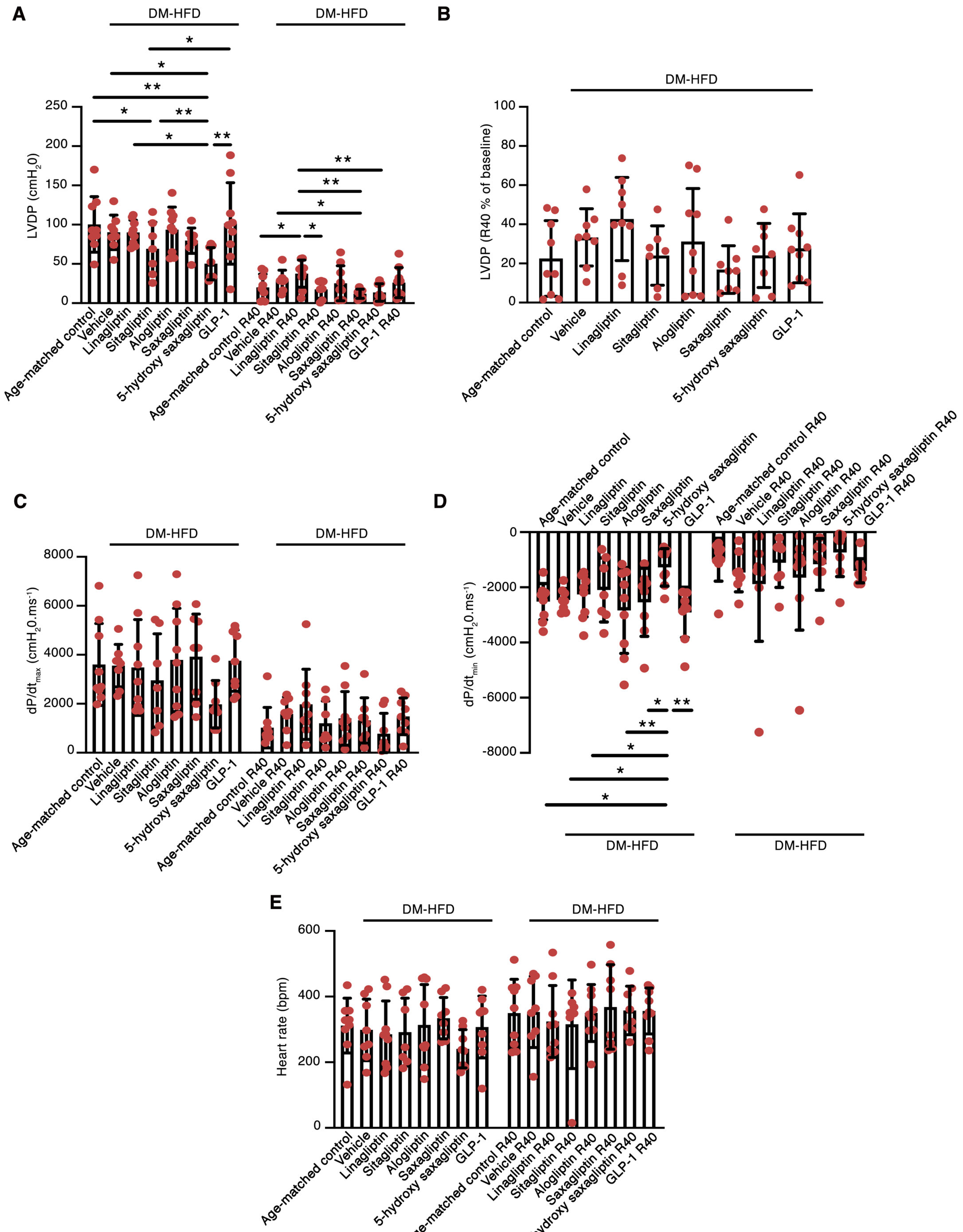

Supporting Information Figure 4. Effect of different DPP-4 inhibitors, administered at human C_{max} equivalent, on the ex vivo function of aged, diabetic high fat diet-fed (DM-HFD) female mice at baseline and 40 minutes after ischemia perfusion (R40). (A) Left ventricular developed pressure (LVDP). (B) Percentage recovery of LVDP 40 minutes after reperfusion (%LVDP R40). (C) $dPdt_{max}$. (D) $dPdt_{min}$. (E) Heart rate. bpm = beats per minute. Values are mean ± S.D.. Age-matched control (n=8), vehicle (n=7), linagliptin (n=9), sitagliptin (n=7), alogliptin (n=8), saxagliptin (n=7), 5-hydroxy saxagliptin (n=7), GLP-1 (n=9). *P < 0.05, **P < 0.01 by 1-way ANOVA followed by Fisher least significant difference post hoc test.

Supplementary Information Table 1. Differentially expressed (upregulated) genes in control cardiomyocytes exposed to linagliptin (9nmol/L) for 24h in comparison to vehicle (n=5/condition).

Gene name	log2(Fold change)	Fold change	p_value	q_value
Pde2a	1.123765923	2.179150629	0.026728817	0.788844993
Pakap	1.097769341	2.140235188	0.002002104	0.626341967
Itgav	0.841945222	1.792465339	0.046965774	0.850540672
Tmem230	0.704445688	1.629531092	0.023037313	0.766040657
Fbxo33	0.667180076	1.587966058	0.006254718	0.668087463
Gm27021	0.633295634	1.551104229	0.004971577	0.6576427
AL732309.1	0.621255347	1.53821306	0.021027694	0.758274182
Crispld2	0.597129679	1.512703963	0.01977031	0.74928387
Arpc5	0.58729635	1.502428516	0.049018486	0.850540672

Supplementary Information Table 2. Differentially expressed (downregulated) genes in control cardiomyocytes exposed to linagliptin (9nmol/L) for 24h in comparison to vehicle (n=5/condition).

Gene name	log2(Fold change)	Fold change	p_value	q_value
Usp50	-1.219344766	0.429477731	0.040243182	0.846390493
Gm27029	-0.866475682	0.548485094	0.00463147	0.6576427
Tmem70	-0.853630319	0.553390461	0.048466767	0.850540672
AC158777.2	-0.811218683	0.569900245	0.035967721	0.822769655
Gm45837	-0.753195136	0.593288146	0.039033096	0.833688523
Ccdc71	-0.638440549	0.64240697	0.04315159	0.849313459
Ninj1	-0.631371081	0.645562606	0.026121056	0.786597241
AK157302	-0.613784245	0.653480348	0.027939	0.790632397
Lmo2	-0.596327165	0.661435707	0.036328525	0.822769655

Supplementary Information Table 3. Differentially expressed (upregulated) genes in cardiomyocytes isolated from diabetic high fat diet-fed mice exposed to linagliptin (9nmol/L) for 24h in comparison to vehicle (n=3/condition).

Gene name	log2(Fold change)	Fold change	p_value	q_value
Scamp4	1.851557641	3.608896181	0.005031597	0.998619142
Myh7	1.361132615	2.568867742	0.019358164	0.998619142
Tmem70	1.336209206	2.524870171	0.00502486	0.998619142
ALT732309.1	1.282451651	2.432519973	0.004318908	0.998619142
Ramp2	1.050820552	2.071707824	0.007812122	0.998619142
Paqr7	0.984806517	1.97904788	0.025137932	0.998619142
Gcdh	0.83411352	1.782761264	0.017684976	0.998619142
Umad1	0.821971132	1.767819688	0.010382348	0.998619142
Pigq	0.812556816	1.756321326	0.049406831	0.998619142
Cbfa2t	0.70972981	1.63549779	0.036951267	0.998619142
Plpp7	0.680145043	1.602300836	0.015728579	0.998619142
Cuedc1	0.656655018	1.576423343	0.037036577	0.998619142
Phrf1	0.644183357	1.562854376	0.006744636	0.998619142
Lbhd1	0.632895933	1.550674553	0.02958863	0.998619142
Thra	0.621814976	1.538809857	0.016168957	0.998619142
Amdhd2	0.620319757	1.53721585	0.018407393	0.998619142
Selenbp1	0.603192739	1.519074619	0.039961717	0.998619142
Lysmd1	0.589447986	1.50467091	0.035437357	0.998619142

Supplementary Information Table 4. Differentially expressed (downregulated) genes in cardiomyocytes isolated from diabetic high fat diet-fed mice exposed to linagliptin (9nmol/L) for 24h in comparison to vehicle (n=3/condition).

Gene name	log2(Fold change)	Fold change	p_value	q_value
Adat3	-1.615436324	0.326366227	0.000189328	0.978868628
Slc10a4	-1.204130317	0.434030907	0.030403769	0.998619142
Itgb6	-0.651098367	0.636795318	0.013982245	0.998619142
Ins16	-0.636795318	0.64444906	0.005209072	0.998619142
Esyt2	-0.587954972	0.665285284	0.007292278	0.998619142
